# Redefining Chemoresistance: Natural Bioactives as Molecular Modulators at the Cancer–Tumor Microenvironment Interface

**DOI:** 10.3390/ijms26168037

**Published:** 2025-08-20

**Authors:** Claudia Reytor-González, Emilia Jiménez-Flores, Natalí González, Daniel Simancas-Racines

**Affiliations:** 1 Universidad UTE, Facultad de Ciencias de la Salud Eugenio Espejo, Centro de Investigación en Salud Pública y Epidemiología Clínica (CISPEC), Quito 170527, Ecuador; claudia.reytor@ute.edu.ec (C.R.-G.); mariae.jimenez@ute.edu.ec (E.J.-F.); 2 Universidad UTE, Facultad de Odontología, Quito 170527, Ecuador; natali.gonzalez@ute.edu.ec

**Keywords:** cancer, chemoresistance, bioactive compounds, tumor microenvironment, healthcare

## Abstract

Therapeutic resistance remains a critical barrier in effective cancer treatment, contributing to disease recurrence, progression, and reduced patient survival. In recent years, natural bioactive compounds have emerged as promising adjuncts in oncology due to their ability to modulate multiple biological processes involved in resistance. This review explores current evidence on the role of natural compounds in influencing cancer cell behavior and their interactions with the tumor microenvironment. By organizing these compounds into chemical families, we provide a structured overview of their potential to enhance the efficacy of standard chemotherapy and reduce resistance-related mechanisms. We also highlight innovative strategies, including combination therapies and advanced drug delivery systems, that aim to improve their clinical applicability. Overall, this work underscores the relevance of integrating natural bioactives into modern cancer therapy and calls for further translational research to bridge preclinical findings with clinical implementation.

## 1. Introduction

Therapeutic resistance remains one of the most pressing challenges in modern oncology, representing a critical barrier to the long-term success of cancer treatments [1]. Despite significant advances in drug development, molecular diagnostics, and targeted therapies, resistance to chemotherapy continues to limit patient survival and quality of life [2,3]. In both solid and hematologic malignancies, tumor cells frequently adapt to cytotoxic stress through a myriad of mechanisms, ultimately rendering standard chemotherapeutic regimens ineffective [4]. These mechanisms include increased drug efflux, enhanced deoxyribonucleic acid (DNA) repair capacity, apoptosis evasion, metabolic reprogramming, and epigenetic alterations [5]. However, recent insights suggest that the development of chemoresistance is not solely an intrinsic property of cancer cells, but also profoundly influenced by the complex and dynamic ecosystem in which these cells reside, the tumor microenvironment (TME) [6].

The TME, a highly heterogeneous and interactive network composed of stromal cells, immune cells, extracellular matrix (ECM) components, cytokines, and growth factors, plays a pivotal role in shaping cancer progression and therapeutic response [7]. This microenvironment not only facilitates tumor growth and invasion but also actively participates in the development of resistance through paracrine signaling, immune modulation, and the induction of phenotypic plasticity in tumor cells [8]. The bidirectional crosstalk between cancer cells and the TME fosters a milieu conducive to survival under therapeutic pressure, thereby contributing to both primary and acquired chemoresistance [9].

Considering the limitations of conventional monotherapies and the inherent complexity of resistance pathways, there is a growing interest in agents capable of simultaneously targeting multiple molecular and cellular processes [10]. Natural bioactive compounds, secondary metabolites derived from plants, fungi, bacteria, and marine organisms, have emerged as promising candidates in this regard [11,12,13,14]. Unlike synthetic drugs, which often act on a single target or pathway, many natural compounds possess broad-spectrum biological activities that enable them to modulate a wide array of signaling cascades implicated in cancer pathogenesis and treatment resistance [15].

Notably, several natural bioactives such as curcumin, resveratrol, quercetin, epigallocatechin gallate (EGCG), and sulforaphane have demonstrated the ability to interfere with key regulators of chemoresistance, including nuclear factor-kappa B (NF-κB), phosphoinositide 3-kinase/protein kinase B (PI3K/Akt), signal transducer and activator of transcription (STAT) 3, and hypoxia-inducible factors (HIFs) [16]. These compounds also exhibit anti-inflammatory, antioxidant, anti-angiogenic, and immunomodulatory properties, positioning them as ideal candidates for disrupting the protective mechanisms conferred by the TME [17]. In preclinical studies, many of these bioactives have been shown to sensitize resistant tumor cells to chemotherapeutic agents, reverse multidrug resistance phenotypes, and attenuate the pro-tumorigenic effects of stromal elements [18,19,20].

Given their pleiotropic nature, relatively low toxicity, and potential for synergistic interactions with standard therapies, natural bioactives are increasingly being investigated as adjuncts to existing cancer treatment protocols [21]. However, despite promising in vitro and in vivo findings, their clinical translation has been hampered by issues related to bioavailability, pharmacokinetics, and standardization [22]. Nevertheless, advancements in formulation technologies, such as nanoencapsulation and targeted delivery systems, are helping to overcome these limitations, opening new avenues for the integration of natural compounds into evidence-based oncology [23,24,25].

This review aims to synthesize current knowledge on the molecular mechanisms through which natural bioactive compounds influence chemoresistance, with a particular focus on their interactions at the cancer–TME interface. By exploring how these agents modulate key signaling pathways, cellular cross-talk, and environmental stress responses, we seek to provide a comprehensive framework for understanding their potential as multifaceted therapeutic tools. Ultimately, redefining chemoresistance through the lens of natural bioactives may offer novel strategies for improving treatment outcomes and overcoming one of the most formidable obstacles in cancer therapy.

## 2. Key Molecular Mechanisms Underlying Therapeutic Resistance

Tumor drug resistance arises from a complex interplay of cellular adaptations that enable cancer cells to survive even under aggressive therapeutic pressures. One of the most pivotal strategies employed by malignant cells is the evasion of apoptosis. Under normal physiological conditions, chemotherapeutic agents aim to induce programmed cell death via the intrinsic mitochondrial pathway. This involves mitochondrial outer membrane permeabilization, which initiates cytochrome c release and caspase cascade activation, ultimately leading to apoptosis. However, in resistant tumors, this pathway is frequently disrupted. Cancer cells often overexpress anti-apoptotic genes and proteins, which inhibit mitochondrial outer membrane permeabilization and prevent the apoptotic process. Simultaneously, key pro-apoptotic proteins are downregulated or mutated, effectively shifting the balance toward cell survival.

Oncogenes contribute further to this apoptotic evasion. A prime example is Astrocyte Elevated Gene 1, which drives resistance by promoting the translation of multidrug resistance protein 1 (MDR1) mRNA, thereby enhancing efflux of chemotherapeutics such as doxorubicin. This oncogene also activates adenosine monophosphate-activated protein kinase (AMPK)/mammalian target of rapamycin (mTOR) signaling, initiating autophagy as a protective mechanism and mediating resistance to doxorubicin and 5-fluorouracil. Furthermore, it impairs retinoic acid receptor function and upregulates MET receptor tyrosine kinase and Aldehyde Dehydrogenase 3 Family Member A1, reinforcing resistance through multiple molecular axes [26]. Another oncogene implicated in chemoresistance is AXL receptor tyrosine kinase, frequently overexpressed in resistant tumors. It facilitates resistance through its interaction with downstream receptor tyrosine kinase (RTK) signaling pathways, including Epidermal Growth Factor Receptor (EGFR), human epidermal growth factor receptor (HER) 2, and 3, thus contributing to enhanced cell survival and adaptation [27].

Survival pathways activated downstream of RTKs further entrench resistance. The PI3K/AKT/mTOR and Mitogen-Activated Protein Kinase (MAPK) signaling cascades are central to these processes. PI3K, upon activation, catalyzes the formation of Phosphatidylinositol-3,4,5-trisphosphate, which recruits and activates AKT via phosphorylation by 3-phosphoinositide-dependent protein kinase-1 and mTORC (mammalian target of rapamycin complex) 2. Activated AKT promotes cell survival by inactivating pro-apoptotic targets such as B-cell lymphoma (BCL) 2-Associated agonist of cell death and caspase-9, while simultaneously activating mTORC1, which enhances protein synthesis and cellular proliferation. In parallel, the MAPK pathway activates nuclear transcription factors like E-twenty-six-like transcription factor 1 and cellular oncogene fos, leading to upregulation of cyclin D1 and anti-apoptotic proteins, including BCL-2 and myeloid cell leukemia (MCL) -1. The Janus kinase (JAK)/STAT pathway complements this network. Activated STAT3 and STAT5 transcriptionally induce anti-apoptotic and immunosuppressive genes, facilitating both tumor survival and immune evasion. NF-κB, another key signaling axis, is activated through IκB kinase degradation and supports tumor persistence through the transcription of pro-survival and pro-inflammatory genes. These pathways are not isolated; extensive crosstalk among them ensures signal redundancy and robustness, complicating therapeutic efforts. PI3K/AKT can amplify NF-κB signaling, while MAPK and JAK/STAT share transcriptional targets with overlapping biological outcomes [28].

In tandem with these survival networks, dysregulated autophagy plays a substantial role in resistance. Autophagy, a lysosome-mediated degradation process, allows cells to recycle damaged components and generate metabolic substrates necessary for energy production and redox balance. In cancer, autophagy is frequently upregulated under therapeutic stress, enabling cells to avert apoptosis and sustain their bioenergetic demands. Autophagy initiation is controlled by the Unc-51-like autophagy activating kinase 1 complex, inhibited by mTORC1 under nutrient-rich conditions. Inhibition of mTORC1, via starvation or targeted drugs, releases this complex, initiating autophagosome formation with the help of Vacuolar Protein Sorting 34 and Beclin-1. While mTOR inhibition may impede proliferation, it simultaneously triggers autophagy, creating a paradox in treatment strategies. Extracellular signal-regulated kinases (ERKs) 1 and 2 in the MAPK pathway also contribute by modulating autophagy-related proteins and transcription factors, reinforcing cell survival under stress [29].

**Figure 1:**
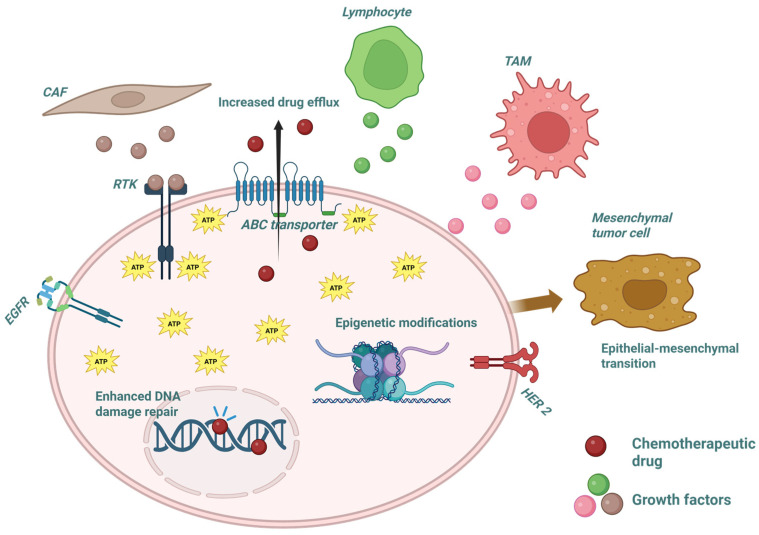
Pathways underlying cancer drug resistance.

One of the most prominent and well-characterized mechanisms of drug resistance involves increased drug efflux via ATP-binding cassette (ABC) transporters (Figure 1). Among these, P-glycoprotein (P-gp)/ATP-binding cassette sub-family B member 1 (ABCB1) is the most well-known and frequently implicated in multidrug resistance (MDR). P-gp plays a physiological role in protecting tissues by pumping out xenobiotics and toxins, but in tumors, its overexpression contributes to chemotherapy failure. Notably, its regulation is influenced by transcriptional inputs from PI3K/AKT and NF-κB signaling pathways. Additionally, epigenetic alterations, such as hypermethylation of the ABCB1 promoter, can paradoxically increase P-gp expression [30].

Other transporters such as ABCG2 and ABCC1 also participate in MDR, handling a broad spectrum of anticancer drugs including anthracyclines, taxanes, and platinum compounds [31]. ABCG2, for instance, acts as a xenobiotic pump with a significant role in effluxing chemotherapeutics in resistant cancers [32]. These ABC proteins are not only expressed on the plasma membrane, but also in intracellular vesicles, and they critically influence the pharmacokinetics of anticancer agents in humans. Importantly, they are also found in extracellular vesicles, which participate in drug expulsion and the horizontal transfer of resistance phenotypes to other cells in the TME [33]. Across solid tumors, overexpression of ABC transporters, particularly P-gp, remains one of the most consistent features of acquired drug resistance [30].

DNA repair mechanisms and epigenetic modifications further enhance resistance (Figure 1). DNA methyltransferases (DNMTs) catalyze the addition of methyl groups to cytosine residues in cytosine–phosphate–Guanine islands, silencing the expression of tumor suppressor genes such as tumor protein p53 and pro-apoptotic effectors like BAX. Meanwhile, histone deacetylases (HDACs) promote chromatin compaction and gene repression, counteracted by histone acetyltransferases that facilitate gene transcription through chromatin relaxation. These changes are not static; they are dynamically regulated by oncogenic signaling pathways and tumor-derived environmental cues. Moreover, non-coding RNAs, including microRNAs and long non-coding RNAs, contribute significantly to resistance by targeting genes involved in apoptosis, drug metabolism, and DNA repair. Dysregulation in the miRNA biogenesis machinery, such as aberrant activity of Drosha and Dicer, further disrupts microRNA profiles, exacerbating resistance. Notably, some microRNAs have been shown to increase P-gp expression, linking epigenetic regulation directly to drug efflux mechanisms [34,35].

Beyond genetic and epigenetic factors, metabolic reprogramming underlies another layer of tumor adaptability. Cancer cells frequently adopt aerobic glycolysis, a phenomenon known as the Warburg effect, in which glucose is converted into lactate despite sufficient oxygen availability, suggesting the beneficial effects of adopting a low-sugar diet [36]. This metabolic shift supports biosynthetic processes and ATP production needed for rapid proliferation. Additionally, mitochondrial adaptations enhance redox balance and bioenergetic flexibility, promoting cell survival even under nutrient-deprived and hypoxic conditions [37].

This schematic outlines key cellular and molecular mechanisms underlying drug resistance, including tumor microenvironment changes, epigenetic alterations, epithelial-to-mesenchymal transition (EMT), enhanced drug efflux, and increased DNA repair. Drug efflux transporters use ATP hydrolysis to expel chemotherapeutic agents (30), while upregulated DNA repair systems enable cancer cells to survive genotoxic therapies such as platinum-based drugs and topoisomerase inhibitors [30,38,39]. Abbreviations—ABC: ATP binding cassette; EGFR: epidermal growth factor receptor; ATP: adenosine triphosphate; DNA: deoxyribonucleic acid; RTK: receptor tyrosine kinases; CAF: cancer-associated fibroblast; TAM: tumor-associated macrophage; and HER 2: human epidermal growth factor receptor 2.

## 3. The Tumor Microenvironment as a Co-Regulator of Resistance

**Figure 2:**
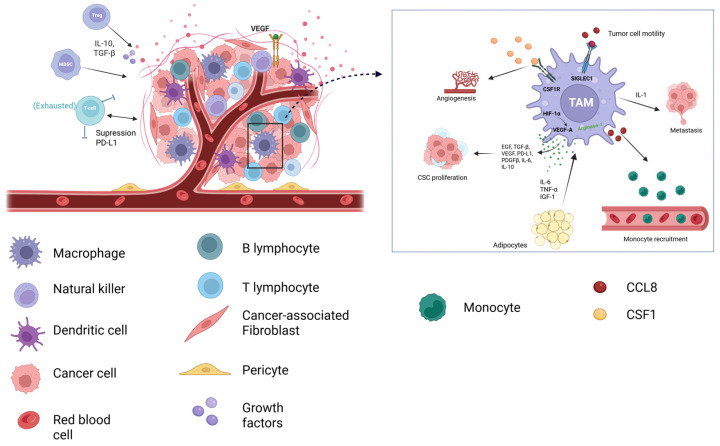
Tumor microenvironment constituents and the contribution of tumor-associated macrophages to cancer progression.

The TME is increasingly recognized as a pivotal co-regulator of cancer progression and therapeutic resistance. Composing a heterogeneous network of malignant cells, stromal components, immune infiltrates, and ECM, the TME forms a dynamic ecosystem that profoundly influences tumor biology (Figure 2) [40].

At the core of tumor heterogeneity lies the presence of cancer stem cells (CSCs), a subpopulation endowed with self-renewal, differentiation capacity, and tumor-initiating potential [41]. These cells orchestrate tumor relapse and drug resistance through intrinsic features such as quiescence, enhanced DNA damage response, and overexpression of ABC transporters [39]. Molecularly, CSCs engage signaling pathways including wingless-type MMTV integration site family (Wnt)/β-catenin, Notch, Hedgehog, PI3K/AKT, and NF-κB, which promote survival, stemness, and inflammatory microenvironments conducive to therapy evasion [42]. Physiologically, CSCs often reside in specialized hypoxic niches within the tumor, where HIFs, primarily HIF-1α, mediate metabolic reprogramming to glycolysis and autophagy, supporting CSC maintenance and resistance [42]. Hypoxic stress also induces EMT, enhancing CSC invasiveness and metastatic potential [43]. Together, these molecular and environmental adaptations empower CSCs to survive cytotoxic assaults and contribute substantially to intratumoral heterogeneity and therapeutic failure [43].

Tumor-associated macrophages (TAMs) represent a key immunosuppressive cell population within the TME, arising from circulating monocytes recruited and polarized by tumor-derived signals (Figure 2) [44]. Recent studies have elucidated mechanisms underlying TAM-mediated resistance, including the polarization of macrophages via signaling pathways such as STAT3/IL-10 and PI3K/AKT, which enhance the efficacy of immune checkpoint blockade when modulated [45,46,47]. Moreover, TAMs interplay with adipocytes within the TME, wherein adipocyte-secreted inflammatory cytokines activate NF-κB signaling in macrophages, linking obesity-associated inflammation to endocrine therapy resistance [48,49]. Effective management of obesity is essential, as the chronic inflammation associated with this condition has been implicated in the development or aggravation of various diseases, including cancer [50,51,52,53,54,55].

Cancer-associated fibroblasts (CAFs), the predominant stromal cell type in many solid tumors, originate from activated fibroblasts influenced by tumor-secreted cytokines such as transforming growth factor beta (TGF-β), epidermal growth factor (EGF), platelet-derived growth factor (PDGF), and fibroblast growth factor (FGF) 2, and can also derive from mesenchymal stem cells or other cell types via transdifferentiation [56]. These cells modulate tumor progression and resistance through diverse mechanisms, including remodeling the ECM, secreting growth factors and cytokines, and mediating metabolic crosstalk [57]. CAFs enhance CSC survival by activating pathways such as Wnt/β-catenin, NF-κB, and STAT3, thus promoting stemness and resistance to agents like oxaliplatin, 5-fluorouracil, gemcitabine, and cisplatin in colorectal, gastric, and pancreatic cancers [58,59,60]. Exosomes secreted by CAFs carry microRNAs and proteins that modulate tumor cell gene expression, induce autophagy, and facilitate drug resistance [61,62,63,64,65,66]. CAFs also regulate immune suppression by recruiting immunosuppressive cells and inducing their polarization via cytokines such as IL-6, TGF-β, and Insulin-like Growth Factor Binding Protein 7, further blunting anti-tumor immunity [57].

A notable CAF function is their involvement in promoting resistance to radiotherapy [67]. They do so by secreting protective cytokines (e.g., IL-6, TGF-β), facilitating autophagy in cancer cells, and direct cell contact, activating survival pathways such as β1 integrin and Focal Adhesion Kinase (FAK) signaling [42]. CAFs are intrinsically radioresistant, partly due to defects in p53/p21 and high expression of Survivin, and radiotherapy itself can induce a secretory phenotype in CAFs characterized by pro-tumorigenic cytokines like IL-6, IL-8, and osteopontin that enhance tumor proliferation and immunosuppression post-irradiation [57]. Additionally, CAFs upregulate immune checkpoint molecules in cancers such as colorectal and lung adenocarcinoma, promoting immune escape [68,69]. They also contribute to ECM remodeling and aberrant angiogenesis, limiting immune cell infiltration and fostering an immunosuppressive milieu [70,71,72,73].

Endothelial cells and cancer-associated adipocytes (CAAs), though less abundant than CAFs, are critical in shaping tumor progression and therapy resistance. Tumor endothelial cells facilitate aberrant neovascularization and vasculogenesis, which can exacerbate hypoxia and promote resistance by limiting T cell infiltration [57]. Vascular endothelial growth factor (VEGF), secreted by endothelial and tumor cells, is central to angiogenesis and suppresses dendritic cell maturation, reducing antigen presentation and enhancing programmed cell death 1 ligand 1 (PD-L1) expression on immune cells, thereby impairing effective T cell responses [74]. ECM remodeling is exacerbated by hypoxia and HIF-1α signaling, which upregulate collagen and fibrosis-associated genes, further reinforcing an immunosuppressive and pro-metastatic niche [75]. Enzymes like lysyl oxidase cross-link ECM fibers, increasing tumor stiffness and acting as a physical barrier to therapeutics [76]. Additionally, aberrant ECM contributes to metabolic stress and activates integrin-FAK signaling pathways in tumor cells, promoting survival and therapy resistance [77].

Hypoxia, a hallmark of solid tumors driven by inadequate vascularization relative to rapid tumor growth, activates HIFs, mainly HIF-1α, which orchestrate transcriptional programs that regulate angiogenesis, metabolic adaptation, immune modulation, and ECM remodeling [76]. Stabilized HIF-1α induces proangiogenic factors such as VEGF, PDGF, FGF, angiopoietins, and matrix metalloproteinases (MMPs), driving the formation of abnormal, leaky vasculature that facilitates invasion, metastasis, and therapy resistance [78,79]. Additionally, HIF-1α modulates metabolic reprogramming toward glycolysis, generating acidic microenvironments rich in lactate that suppress immune effector cells while promoting regulatory populations like regulatory T cells and myeloid-derived suppressor cells (MDSCs) [42]. Reactive oxygen species (ROS) and endoplasmic reticulum stress further regulate HIF signaling, adding layers of complexity to tumor adaptation [80]. The temporal “HIF switch” from HIF-1α in acute hypoxia to HIF-2α in chronic states enables sustained vascular maturation and energy balance, reinforcing tumor progression [81]. Hypoxia also induces immune checkpoints such as PD-L1, Human Leukocyte Antigen-G, CD47, and V-domain Ig Suppressor of T cell Activation, which inhibit T cell and macrophage function and facilitate immune evasion [42].

Collectively, the interplay among CSCs, TAMs, CAFs, endothelial cells, CAAs, and the ECM under hypoxic and immunosuppressive conditions drives tumor heterogeneity, immune escape, angiogenesis, and resistance to diverse therapies [82]. These reciprocal interactions and adaptive mechanisms underscore the complexity of targeting the TME to overcome resistance. While targeting single components like immune checkpoints or angiogenesis has yielded limited success, integrative strategies that consider the multifactorial nature of TME regulation are essential for improving clinical outcomes. Emerging technologies such as single-cell RNA sequencing hold promise for unraveling stromal-immune crosstalk and identifying novel therapeutic targets [57].

Lastly, emerging evidence highlights the role of gut microbiota in oncogenesis and therapy resistance, particularly in colorectal cancer [83]. Dysbiosis, characterized by the predominance of colibactin-producing *Escherichia coli*, induces DNA damage, ROS generation, and chromosomal instability, contributing to tumor progression and chemoresistance. Targeting such microbial factors may represent novel adjunctive strategies in cancer treatment [84,85].

The TME drives tumor heterogeneity, immune escape, and resistance to chemotherapy, radiotherapy, and immunotherapy. Stromal fibroblasts, immune cells, ECM components, and vasculature activate pro-survival signaling in tumor cells, remodel the ECM to hinder drug delivery, and promote VEGF-independent vascularization. CAAs secrete IL-6, tumor necrosis factor alpha (TNF-α), and insulin-like growth factor-1, stimulating proliferative and survival pathways, metabolic reprogramming, and chronic inflammation through the autotaxin–lysophosphatidic acid pathway. Tumor cells evade immune surveillance by downregulating tumor-associated antigens and major histocompatibility complex molecules, overexpressing immune checkpoint ligands, shedding antigens, and releasing immunosuppressive mediators such as TGF-β, IL-10, and PD-L1, resulting in cytotoxic T lymphocyte exhaustion and tumor persistence. TAMs further contribute to tumor progression, metastasis, angiogenesis, invasion, and CSC expansion through cytokine and growth factor release. CCL8 recruits monocytes, induces CSF-1 production to maintain TAM survival and proliferation, and binds SIGLEC1 to enhance tumor cell motility; inhibition of the CSF-1/CSF-1 receptor axis reduces angiogenesis. IL-1 facilitates metastatic cell recruitment and colonization. Due to their plasticity, TAMs exhibit distinct phenotypes: M1-like TAMs mediate tumoricidal, pro-inflammatory effects, whereas M2-like TAMs promote tumor growth, invasion, angiogenesis, and immune suppression via IL-10, TGF-β, VEGF, and PD-L1. M2-like TAMs also enhance therapeutic resistance by protecting tumor cells from immune destruction, inducing abnormal vasculature, and inhibiting CTL activity [86,87,88,89,90,91,92,93,94,95,96,97,98,99]. Abbreviations—TME: tumor microenvironment; ECM: extracellular matrix; CAAs: cancer-associated adipocytes; MHC: major histocompatibility complex; CCL8: C-C motif chemokine ligand 8; MDSC: myeloid-derived suppressor cell; TGF-β: transforming growth factor beta; Treg: regulatory T cell; PD-L1: programmed cell death 1 ligand 1; VEGF: vascular endothelial growth factor; TAM: tumor-associated macrophage; SIGLEC1: sialic acid-binding immunoglobulin-type lectin 1; IL-1: interleukin-1; IL-6: interleukin-6; IL-10: interleukin-10; TNF– α: tumor necrosis factor-alpha; IGF-1: insulin-like growth factor-1; EGF: epidermal growth factor; PDGFβ: platelet-derived growth factor beta; CSC: cancer stem cell; CSF1: colony-stimulating factor-1; CSF1R: colony-stimulating factor 1 receptor; CTL: cytotoxic T lymphocytes; HIF-1α: hypoxia-inducible factor 1-alpha; and VEGF-A: vascular endothelial growth factor A.

## 4. Natural Bioactive Compounds with Functional Evidence

**Figure 3:**
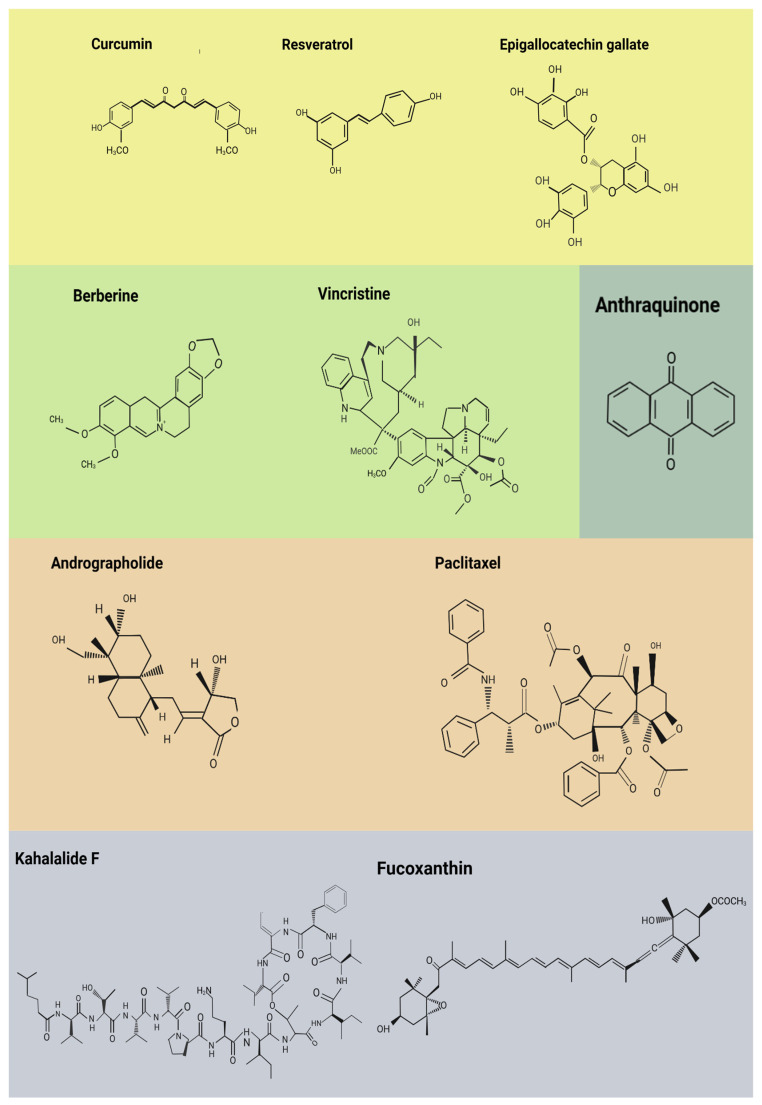
Chemical structures of natural compounds. This figure showcases the chemical structures of bioactive natural products, including polyphenols (yellow), alkaloids (green), terpenoids and diterpenes (light brown, bioactive lipids and marine-derived metabolites (light grey, and anthraquinones (turquoise). These compounds, sourced from terrestrial and marine environments, exhibit a wide range of biological activities such as antioxidant, anti-inflammatory, antimicrobial, and anticancer effects, reflecting the chemical diversity and therapeutic potential of natural substances [115,116,117,118,119,120,121,122,123].

**Figure 4:**
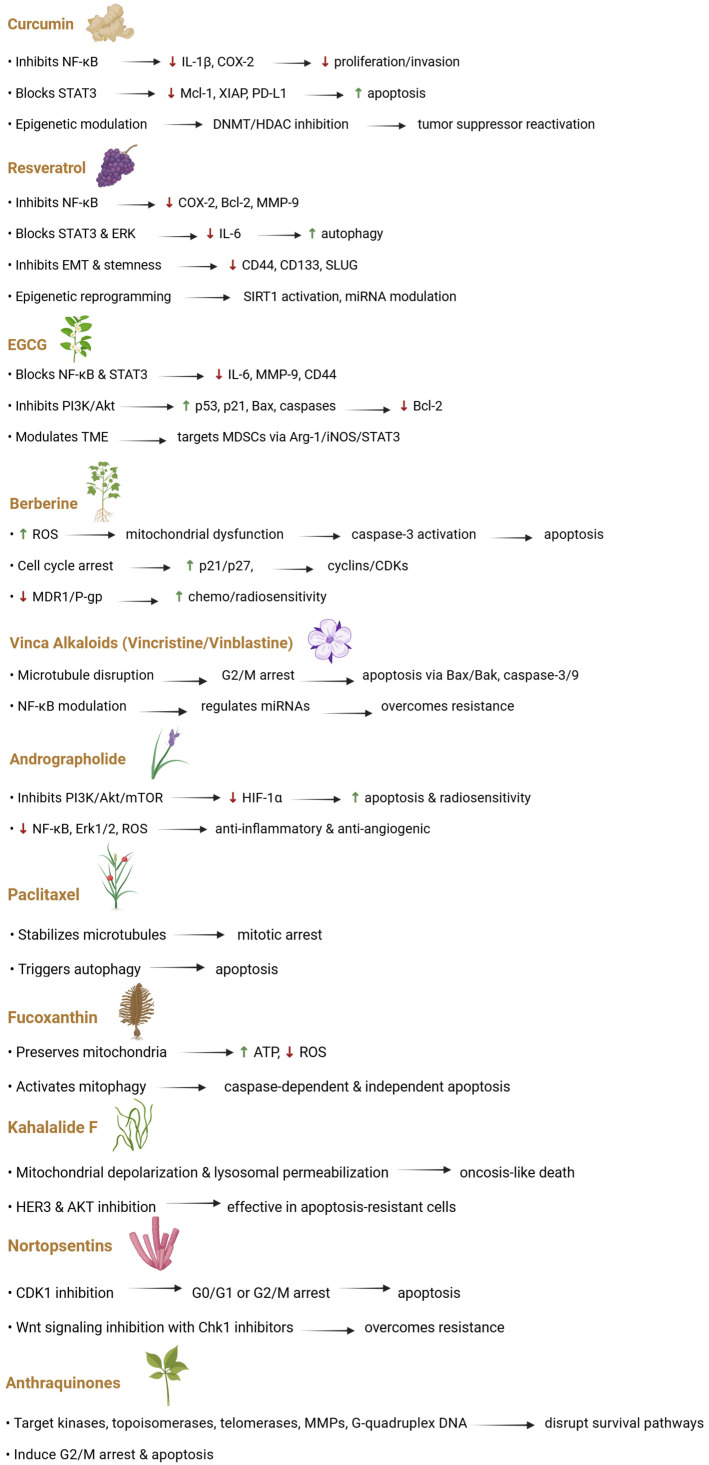
Anticancer mechanisms and resistance pathways of natural bioactive compounds. Natural bioactives and their molecular actions, including inhibition of oncogenic signaling, modulation of inflammatory pathways, induction of apoptosis and cell cycle arrest, epigenetic regulation, activation of autophagy, and targeting of the tumor microenvironment. Several compounds also enhance therapeutic sensitivity and counteract multidrug resistance [115,124,125,126,127,128,129,130,131,132,133,134,135,136]. Green arrows indicate an enhanced effect; red arrows indicate a diminished effect. Abbreviations—Akt: protein kinase B; Arg-1: arginase-1; ATP: adenosine triphosphate; Bak: Bcl-2 homologous antagonist killer; Bax: Bcl-2-associated X protein; Bcl-2: B-cell lymphoma 2; CD44: cluster of differentiation 44; CD133: cluster of differentiation 133; CDKs: cyclin-dependent kinases; Chk1: checkpoint kinase 1; COX-2: cyclooxygenase-2; DNA: deoxyribonucleic acid; DNMT: DNA methyltransferase; EGCG: epigallocatechin gallate; Erk1/2: extracellular signal-regulated kinase ½; HDAC: histone deacetylase; HER3: human epidermal growth factor receptor 3; HIF-1α: hypoxia-inducible factor 1-alpha; IL-1β: interleukin-1 beta; IL-6: interleukin-6; iNOS: inducible nitric oxide synthase; Mcl-1: myeloid cell leukemia 1; MDR1: multidrug resistance protein 1; MDSCs: myeloid-derived suppressor cells; miRNA: microRNA; MMP: matrix metallopeptidase; mTOR: mammalian target of rapamycin; NF-κB: nuclear factor kappa-light-chain-enhancer of activated B cells; P-gp: P-glycoprotein; PD-L1: programmed death-ligand 1; p21: cyclin-dependent kinase inhibitor 1; p27: cyclin-dependent kinase inhibitor 1B; p53: tumor protein p53; PI3K: phosphoinositide 3-kinase; ROS: reactive oxygen species; SIRT1: sirtuin 1; SLUG: Zinc finger protein SNAI2; STAT3: signal transducer and activator of transcription 3; TME: tumor microenvironment; Wnt: wingless-related integration site; and XIAP: X-linked inhibitor of apoptosis protein.

Since the interconnected mechanisms described above form a strong barrier to effective cancer treatment, there is growing interest in combination therapies that target multiple pathways, such as autophagy, epigenetic alterations, drug efflux, and dysbiosis, to overcome resistance and improve patient outcomes [100]. Emerging nutritional strategies, including calorie restriction and ketogenic or plant-based diets (i.e., Mediterranean diet), aim to disrupt cancer metabolism and counteract resistance, supporting their use as valuable adjuncts in oncology and therapeutic approaches for other conditions [101,102,103,104,105,106,107,108,109]. These effects are largely attributed to the rich content of bioactive compounds in such diets [110,111], including polyphenols, alkaloids, terpenoids, diterpenes, bioactive lipids, marine-derived metabolites and anthraquinones (Figure 3), which modulate key pathways such as inflammation, oxidative stress, DNA repair, and apoptosis (Figure 4) [112,113,114].

### 4.1. Polyphenols

Polyphenols represent a broad spectrum of plant-based secondary metabolites recognized for their ability to regulate oxidative balance, immune responses, and carcinogenic pathways [137]. Beyond their well-established antioxidant roles, these compounds modulate inflammation, promote apoptosis, and influence epigenetic and transcriptional landscapes. Their biological activities are often mediated through signaling cascades involving NF-κB, STAT3, PI3K/Akt, and MAPK, which are frequently dysregulated in cancer [138,139,140].

Curcumin, a non-flavonoid polyphenol from *Curcuma longa*, exerts potent anti-inflammatory and anticancer effects through diverse molecular targets. It inhibits NF-κB signaling by stabilizing the inhibitor of nuclear factor kappa B alpha and blocking the nuclear translocation of NF-κB subunits, thereby downregulating pro-inflammatory mediators such as IL-1β and Cyclooxygenase (COX)-2 [141]. In breast cancer cells, it reduces p100/p52 expression, limiting proliferation and invasion [142]. Additionally, curcumin disrupts STAT3 activation by preventing its phosphorylation and DNA-binding activity, suppressing the expression of survival genes like Mcl-1, X-linked inhibitor of apoptosis protein gene, and PD-L1 [143,144,145]. It also enhances antitumor immunity by increasing CD8^+^ T cell infiltration and modulating the TME [146].

On an epigenetic level, curcumin inhibits DNMTs and HDACs, alters histone modifications, and restores tumor suppressor gene expression [147,148,149]. It also regulates a broad array of non-coding RNAs, particularly microRNAs and long non-coding RNAs, which influence cancer cell proliferation, invasion, metastasis, and apoptosis in several tumor types, including breast, gastric, ovarian, and colorectal cancers (MING). Through this modulation, curcumin can reverse chemoresistance, enhancing the efficacy of conventional anticancer therapies [150].

Resveratrol, another polyphenol found in grapes and berries, is known for its capacity to inhibit cancer progression by disrupting NF-κB-mediated transcription of inflammatory and anti-apoptotic genes, including COX-2, Bcl-2, and MMP-9 [151,152,153]. It also targets STAT3 and ERK pathways, downregulating IL-6 production and delaying tumor growth in pancreatic models [154]. In cholangiocarcinoma, inhibition of STAT3 is associated with enhanced autophagy via BECLIN-1 [155]. Additionally, resveratrol hinders EMT and cancer stemness by downregulating CD44, CD133, and SLUG through modulation of PI3K/Akt and Smad signaling [124,125,156,157]. On the epigenetic level, it activates Sirtuin 1 (SIRT1) gene, reshapes histone acetylation patterns, alters DNA methylation, and modulates microRNAs, collectively reprogramming gene expression linked to cell cycle control and apoptosis [158,159].

EGCG, the primary catechin in green tea, inhibits tumor-promoting pathways by blocking NF-κB and STAT3 activation, thus reducing the transcription of IL-6, MMP-9, and CD44 [160,161,162]. EGCG also suppresses the PI3K/Akt axis, leading to activation of p53, p21, Bax, and caspases, while downregulating anti-apoptotic proteins like Bcl-2 [126]. Importantly, EGCG exerts significant epigenetic control by inhibiting DNMTs and HDACs, reactivating silenced tumor suppressors, and regulating EMT-associated microRNAs [163,164,165]. Furthermore, it alters the tumor immune landscape by targeting MDSCs through modulation of the Arginase 1 (Arg-1)/inducible nitric oxide synthase/STAT3 axis and related signaling pathways [166].

### 4.2. Alkaloids

Alkaloids like berberine (BBR) and vincristine are known for their strong anticancer properties, primarily due to their ability to modulate key intracellular signaling networks that govern angiogenesis inhibition, mitochondrial stability, cell cycle progression, and mechanisms of multidrug resistance [167]. BBR, a naturally occurring benzylisoquinoline alkaloid with a characteristic yellow crystalline appearance, is present in various parts of medicinal plants, including roots, bark, leaves, and rhizomes [168]. On a mechanistic level, BBR initiates apoptosis and mitochondrial impairment by elevating intracellular ROS, prompting cytochrome c release, and activating caspase-3 through the upregulation of Growth Arrest and DNA Damage-inducible protein 153 (GADD153) and the involvement of the PI3K/Akt/mTOR and AMPK–p53 axes [169]. In cancers such as cervical, thyroid, and glioblastoma, it further promotes intrinsic apoptotic signaling, increases intracellular calcium levels, and shifts the Bax/Bcl-2 ratio in favor of apoptosis [170].

BBR also plays a crucial role in halting cell cycle progression at multiple checkpoints (G0/G1, G1, and G2/M), largely by increasing the expression of cyclin-dependent kinase inhibitors CIP1/p21 and Kip1/p27, while downregulating cyclins A and D1 and cyclin-dependent kinases 1, 2, 4, and 6 [171]. These effects are reinforced by the suppression of murine double minute 2, which allows for enhanced stabilization and activation of the p53 protein, along with the regulation of specific microRNAs (miR-23a and miR-214-3p) and interactions with calmodulin [172]. Moreover, BBR has been shown to potentiate the effects of multiple chemotherapeutic agents, including cisplatin, doxorubicin, 5-fluorouracil, niraparib, icotinib, and Osimertinib, and to increase tumor sensitivity to radiotherapy [173]. One major contributor to these effects is its ability to downregulate the expression of the MDR1 gene, which encodes the P-gp efflux pump, a key factor in chemotherapy resistance [174]. Notably, in the setting of Candida albicans infection, BBR takes advantage of a pH-dependent reversal in Mdr1p transporter function, typically an efflux mechanism, to enable its own intracellular accumulation, offering a unique antifungal strategy [175].

Vincristine and vinblastine, two Vinca alkaloids derived from *Catharanthus roseus*, exhibit strong cytotoxic effects by interfering with microtubule formation, thereby impairing mitotic spindle assembly and inducing cell cycle arrest at the G2/M transition via cyclin B1 accumulation [176,177]. This disruption ultimately leads to apoptosis through mitochondrial pathways, involving c-Jun N-terminal kinase-mediated activation of pro-apoptotic proteins such as Bax and Bak, mitochondrial outer membrane permeabilization, and caspase-3 and -9 cleavage [127]. Beyond their antimitotic actions, these compounds influence additional signaling pathways, such as NF-κB signaling by facilitating inhibitor of nuclear factor kappa B alpha degradation, enabling nuclear translocation of NF-κB and transcriptional control over genes related to cell death. Furthermore, vincristine regulates a variety of microRNAs, including miR-155, miR-122, miR-1179, and miR-222-3p, which target critical survival pathways such as PI3K/Akt, MAPK, and JAK/STAT, thereby promoting apoptosis and overcoming resistance in cancer cells [178].

### 4.3. Terpenoids and Diterpenes

Among natural bioactive compounds, terpenoids such as andrographolide and paclitaxel have demonstrated promising therapeutic effects in resistance models, particularly through their roles in autophagy modulation and microtubule dynamics. Andrographolide, a diterpenoid lactone extracted from *Andrographis paniculata*, exhibits diverse pharmacological properties, including anti-inflammatory, anti-diabetic, and anticancer activities [179]. Mechanistically, it interferes with pro-survival autophagic signaling by inhibiting the PI3K/AKT/mTOR pathway and activating the c-Jun N-terminal kinase cascade, thereby reducing HIF-1α expression, a key regulator of tumor progression under hypoxia [180]. This action is associated with mitochondrial dysfunction, as shown by increased p53 expression, an elevated Bax/Bcl-2 ratio, and enhanced radiosensitivity in tumor cells [181]. Furthermore, andrographolide exerts anti-angiogenic and anti-inflammatory effects by downregulating NF-κB, Erk1/2, nicotinamide adenine dinucleotide phosphate oxidase, ROS, and P38 MAPK, while concurrently activating activator protein-1 [182]. Its ability to inhibit oncogenic pathways such as NF-κB, HIF-1, and JAK/STAT has been observed across various cancer types, including lung, breast, ovarian, and colorectal malignancies [182,183,184,185].

Paclitaxel, another diterpenoid of natural origin, was first isolated from the bark of *Taxus brevifolia* and is known for its unique mechanism of stabilizing microtubules [128]. By binding to the β-tubulin subunit and promoting M-loop conformational changes, it enhances lateral protofilament interactions and induces mitotic arrest [186,187]. Additionally, paclitaxel triggers autophagy in various cancers by increasing expression of Beclin-1, Autophagy protein 5, and microtubule-associated protein 1 light chain 3, contributing to both apoptotic and caspase-independent cell death pathways [188,189]. Due to sustainability concerns, alternative production approaches, including biosynthesis and tissue culture, are being actively explored [190].

### 4.4. Bioactive Lipids and Marine-Derived Metabolites

Marine-derived bioactive compounds such as fucoxanthin (FX) and kahalalide F (KF) demonstrate significant promise in modulating cellular responses to mitochondrial dysfunction and non-canonical apoptosis [129]. FX, a carotenoid abundant in brown algae, protects cells from mitochondrial stress by preserving membrane potential, enhancing ATP synthesis, and reducing ROS overproduction through the activation of signaling cascades such as Akt/glycogen synthase kinase 3β/Tyrosine-protein kinase Fyn and Nuclear Factor Erythroid 2-Related Factor 2/Heme Oxygenase-1 [191]. FX counteracts toxin-induced mitochondrial dysfunction, such as that caused by Ochratoxin A, by restoring the activity of respiratory complexes I and III, upregulating mitochondrial genes (ND1, ND5, CO-I), and activating mitochondrial biogenesis via peroxisome proliferator-activated receptor gamma coactivator 1-α and nuclear respiratory factor 1 [192]. Its metabolite fucoxanthinol exhibits superior efficacy in restoring antioxidant defenses and mitophagy markers such as PTEN-induced kinase 1 and Parkin, attenuating both ROS and intrinsic apoptotic signaling via caspase-9, -3, and Bax/Bcl-2 modulation [130,192]. Additionally, FX induces apoptosis-inducing factor-dependent, caspase-independent apoptosis, modulates mitochondrial dynamics, and mitigates endoplasmic reticulum stress-related apoptosis via GADD153/glucose-regulated protein 78 suppression [130,193].

KF, a cyclic depsipeptide originally isolated from *Bryopsis* spp., induces rapid mitochondrial membrane depolarization, lysosomal permeabilization, and endoplasmic reticulum dilation, culminating in oncosis-like, caspase-independent cell death [194,195]. Unlike traditional apoptosis, KF disrupts cellular architecture via swelling, blebbing, and lipid bilayer dissolution, possibly mediated by HER3 interference and AKT pathway suppression [196]. Its selectivity for tumor cells and independence from MDR1 or BCL2 pathways highlight its therapeutic potential in apoptosis-resistant cancers [197,198].

Building on the anticancer effects of marine-derived compounds, Nortopsentins, bis-indolyl alkaloids from marine sponges, demonstrate potent cytotoxic and anti-inflammatory activities. These compounds and their derivatives are active against a broad spectrum of cancer cell lines, including lung, breast, colon, ovarian, pancreatic, liver, bladder, prostate, melanoma, renal, sarcoma, and uterine cancers [131]. Their bis-indole scaffold, often containing an imidazole moiety, allows them to target key signaling pathways by inhibiting cyclin-dependent kinase 1, inducing cell cycle arrest at G0/G1 or G2/M phases, and triggering apoptosis through nuclear condensation and membrane blebbing. In colorectal cancer stem cells, adaptive activation of CD44v6-mediated Wnt signaling can occur, but co-treatment with checkpoint Kinase 1 inhibitors like rabusertib disrupts this pathway and promotes apoptosis in both resistant and non-resistant cell populations, highlighting the versatility of Nortopsentin derivatives in targeting multiple oncogenic mechanisms [194].

### 4.5. Anthraquinones and Cobalamins

Anthraquinones, naturally occurring compounds in medicinal plants, exhibit neuroprotective, anti-inflammatory, anticancer, hepatoprotective, and anti-aging activities, largely through modulation of ROS [132]. They inhibit tumor growth by targeting key molecular pathways and proteins, including kinases, topoisomerases, telomerases, matrix metalloproteinases, and G-quadruplex DNA, disrupting processes critical for cancer cell survival. Certain derivatives, such as 1,3-dihydroxy-9,10-anthraquinones, induce G2/M arrest and apoptosis in cancer cells, while tetracyclic anthraquinone–pyridine hybrids show potent activity against both drug-sensitive and multidrug-resistant leukemia cells, suggesting potential to overcome chemoresistance [133]. Safety concerns persist, as compounds like rhein and emodin have been linked to hepatotoxicity, reproductive toxicity, and developmental defects, and chronic laxative use has been associated with melanosis coli and rare irreversible tissue changes [199]. Complementary strategies, such as cobalamins (C_63_H_88_CoN_14_O_14_P), which are vitamin B_12_ derivatives, can enhance anticancer efficacy by selectively targeting tumor cells and improving delivery; for instance, their use in phthalocyanine-based photodynamic therapy reduces dark toxicity and increases therapeutic performance [200,201].

## 5. Emerging Strategies for Therapeutic Combination

Despite notable advancements in cancer therapies, conventional treatments continue to face significant challenges, including nonspecific cytotoxicity, inadequate tumor targeting, and the recurrent development of resistance to drugs [202]. These limitations often lead to suboptimal therapeutic outcomes, relapse, and treatment failure. To address these concerns, growing attention has been given to integrating natural bioactive compounds, particularly those sourced from plants and marine organisms, into standard cancer therapies [203]. These molecules are recognized for their multitargeting abilities and generally exhibit lower toxicity compared to synthetic drugs [202,204,205]. They can influence immune mechanisms, promoting programmed cell death, hindering tumor cell proliferation, and improving tumor sensitivity to conventional chemotherapy. When used in combination with standard anticancer drugs, they often generate synergistic effects that can mitigate toxicity and help overcome multidrug resistance [206].

Numerous natural compounds have demonstrated enhanced anticancer efficacy when administered alongside chemotherapeutics (Table 1). For instance, EGCG intensifies the effects of cisplatin and paclitaxel by increasing DNA damage and lowering oxidative stress levels [207,208]. Curcumin, widely known for its anti-inflammatory and antineoplastic effects, acts through mechanisms including NF-κB inhibition. Nonetheless, its antioxidant properties may counteract ROS-dependent drugs such as doxorubicin [209]. Even so, curcumin remains effective when co-administered with drugs like paclitaxel, melphalan, and prednisone, especially in gastric cancer and multiple myeloma, owing to its ability to block NF-κB signaling [210]. In fact, adherence to the Mediterranean diet has been associated with protective effects against gastric cancer, largely attributed to the bioactive compounds present in plant-based foods [211].

Several additional compounds work through complementary biological pathways. For example, gambogic acid and solamargine have been shown to increase the impact of cisplatin in non-small-cell lung cancer (NSCLC) by repressing Hedgehog signaling. Similarly, sulforaphane and gefitinib exert comparable effects on this pathway [212]. Hederagenin enhances cancer therapy outcomes by modulating autophagy markers such as microtubule-associated protein light chain 3 I and II. Resveratrol, sourced from grapes, bolsters the therapeutic action of paclitaxel by inhibiting anti-apoptotic proteins like Bcl-2 and survivin through mTOR pathway suppression. Flavonoids such as apigenin boost cisplatin-induced apoptosis and reduce cancer stem cell fractions via p53-mediated mechanisms [213]. Other compounds, including dehydrobruceine B, intensify the effectiveness of platinum-based chemotherapy by inducing mitochondrial oxidative stress [214,215]. Notably, curcumin also exhibits protective effects on healthy tissues during cisplatin treatment in NSCLC, supporting its dual role as a sensitizer and a cytoprotective agent [216].

Despite these promising results, many natural compounds face substantial pharmacokinetic challenges. Their clinical application is hindered by issues like low aqueous solubility, instability in physiological environments, limited absorption, and short systemic circulation, which collectively reduce therapeutic efficacy and necessitate higher, potentially toxic, dosages [217]. To address these drawbacks, a variety of advanced drug delivery technologies have been introduced, such as nanocarriers, liposomes, and targeting systems [218].

Nanoparticles (NPs), generally sized between 1 and 100 nanometers, offer numerous benefits. Their high surface area and small dimensions support improved drug encapsulation, sustained release, and preferential accumulation in tumor tissues via the enhanced permeability and retention effect. Furthermore, they can be surface-modified with targeting molecules, such as antibodies, peptides, or folate, to bind tumor-associated receptors like HER 2, EGFR, the transferrin receptor, and αvβ3 integrins, enhancing targeted delivery and minimizing off-target toxicity [219].

The use of environmentally friendly NPs derived from marine organisms further expands therapeutic possibilities. For example, silver NPs produced from the green alga *Caulerpa taxifolia* prompt apoptosis in lung cancer cells through membrane damage. Similarly, jellyfish-derived gold NPs inhibit the activity of oncogenic kinases like AKT and ERK in HeLa cells. Other cases include copper oxide nanoparticles synthesized by *Rhodotorula mucilaginosa* and chitin–silver hybrids from shrimp shells, which promote apoptosis through Bax and caspase activation while suppressing anti-apoptotic proteins like Bcl-2 and Bcl-xL (JEONG). These marine-based nanocarriers offer both inherent anticancer properties and enhanced drug transport functions.

Liposomes, built from lipid bilayers, represent another key nanocarrier with clinical relevance. They protect encapsulated drugs from degradation and can carry both hydrophobic and hydrophilic agents. PEGylation, modification with polyethylene glycol, prolongs systemic circulation by reducing immune detection, improving drug accumulation at the tumor site [220]. A prominent example is PEGylated liposomal doxorubicin, which demonstrates reduced heart toxicity compared to unencapsulated doxorubicin. Innovations in liposome design have led to tumor-specific targeting. For instance, liposomes conjugated with antibodies against melanoma-associated antigen MAGE-A1 have shown improved delivery of doxorubicin in experimental models [221]. FF-10832, a PEGylated liposomal version of gemcitabine, has shown favorable pharmacokinetics, including prolonged half-life, better tumor uptake, and reduced hematologic side effects [220]. Stimuli-responsive delivery systems represent another frontier. These “smart” carriers are engineered to respond to tumor microenvironmental cues such as low pH, redox gradients, or specific enzymes. pH-sensitive liposomes and enzyme-cleavable polymeric NPs allow precise drug release at tumor sites while sparing healthy tissues [222].

Beyond liposomes, solid lipid Nps, nanoemulsions, and dendrimers have been developed to enhance oral bioavailability and avoid first-pass metabolism. These systems shield active agents from enzymatic degradation and improve uptake by optimizing parameters such as particle size, surface charge, and lipid content [217,222]. Additionally, modifying drug molecules into prodrugs or optimizing their chemical structure can further improve metabolic stability. Emerging studies have also highlighted the role of gut microbiota in modulating drug metabolism and absorption. Microbial enzymes can modify drug molecules through hydrolysis, reduction, or deconjugation, influencing therapeutic activity before systemic circulation begins [223]. The effects of these microbial transformations can vary based on diet, age, antibiotic use, and host genetics, complicating pharmacokinetic predictability. Tailoring treatment strategies according to microbiome profiles could significantly enhance personalized medicine approaches.

Nanotechnology has also transformed immunotherapy by improving the delivery of checkpoint inhibitors, vaccines, and immune modulators [224]. Liposomes and nanoparticles have been utilized to transport agents like anti-programmed cell death protein-1 (PD-1), anti-PD-L1, and anti-cytotoxic T-lymphocyte-associated protein-4, improving their tumor localization and enhancing therapeutic results [225]. Targeted nanocarriers equipped with antibodies have improved drug delivery in melanoma models, although excessive targeting affinity may increase off-target interactions [221].

Additional methods include the use of tumor-targeting peptides that bind specifically to integrins overexpressed in tumor vasculature, enhancing nanocarrier penetration and retention [219]. Multifunctional “theranostic” systems, combining therapeutic and diagnostic components, are emerging as valuable tools for real-time monitoring of treatment distribution and efficacy. These systems seek to personalize drug release both spatially and temporally, reduce off-target exposure, and adapt therapy to individual patient needs.

Finally, oral lipid-based platforms, such as self-emulsifying drug delivery systems, have demonstrated improvements in systemic bioavailability by enhancing lymphatic transport and bypassing liver metabolism. Co-delivery of chemotherapeutic agents with efflux pump blockers or modulators of the tumor microenvironment through these platforms further combats resistance by facilitating higher intracellular drug concentrations.

**Table 1:** Synergic effect between natural compounds and chemotherapeutic drugs.

Type	Natural Compound	Chemotherapy Drug	Tissue and Origin	Mechanism
Polyphenol	Curcumin [226]	Doxorubicin	Breast, tumoral	Inhibits the ATPase function of ABCB4 while leaving its protein expression levels unchanged.
	Resveratrol [227]	5-fluorouracil	Colon, tumoral	Regulates the TNF-β signaling cascade, promotes apoptosis, and inhibits NFκB pathway activation.
	Resveratrol [228]	Cisplatin	Basal alveolar epithelial, tumoral	Triggers apoptosis by influencing autophagy-related cell death mechanisms.
	Urolithin A [229]	Oxaliplatin	Colon, tumoral	Stabilization of p53 and activation of its downstream target genes, leading to control of the cell cycle and suppression of glycolysis.
Alkaloid	Neferine and isoliensinine [230]	Cisplatin	Colon, tumoral	Enhanced cellular absorption of Cisplatin and initiation of mitochondrial apoptosis through a ROS-dependent pathway.
	Berberine [231]	Cisplatin	Ovarian, tumoral	Reduction in cell growth along with the promotion of both apoptosis and necroptosis as forms of cell death.
	Emetine [232]	Cisplatin	Ovarian, tumoral	Decreased cell survival capacity.
	Tetrandrine [233]	Cisplatin	Breast, tumoral	Initiation of apoptosis through a mechanism mediated by ROS.
	Piperlongumine [234]	Doxorubicin	Prostate, tumoral	Inhibitory effect on cell proliferation and promotion of apoptosis, marked by increased levels of cleaved poly (ADP-ribose) polymerase and caspase-3 proteins.
	Piperlongumine [235]	Paclitaxel	Intestinal, tumoral	Cell death triggered by ROS.
Terpenoid	Oridonin [236]	Cisplatin	Bronchial epithelium cell, tumoral	Apoptosis initiation via activation of autophagosomes through the AMPK/Akt/mTOR signaling pathway.
	Borneol [237]	Doxorubicin	Glioma cell, tumoral	Borneol increases the cellular absorption of doxorubicin and stimulates the generation of ROS.
	Vielanin k [238]	Doxorubicin	Breast and mammary cell, tumoral and non-tumoral	Triggering of endoplasmic reticulum stress and mitochondrial apoptosis through the IRE1α-TRAF2-JNK signaling pathway.
	Vielanin P [239]	Doxorubicin	Breast and myelegenous leukemia cell, tumoral	Promotion of doxorubicin buildup by decreasing MRP1 expression through the PI3K/Nrf2 signaling pathway.
	Ginkgolide B [240]	Gemcitabine	Pancreatic cell, tumoral	Inhibition of NFκB activity combined with an enhancement of antiproliferative effects.
	Pachymic acid and dehydrotumulosic acid [241]	Doxorubicin and Cisplatin	Liver, breast, and lung cells, tumoral	Blockage of P-glycoprotein (Pgp) activity leading to increased doxorubicin accumulation and amplification of its biological effects.

## 6. Current Challenges and Future Perspectives

Although bioactive compounds such as curcumin, resveratrol, and EGCG have demonstrated promising synergy with conventional chemotherapeutics in preclinical studies, translating these results to clinical efficacy remains a major hurdle. Pharmacokinetic differences between animal models and humans frequently lead to discrepancies in drug absorption, distribution, metabolism, and excretion, resulting in limited clinical success [242,243]. Curcumin, in particular, suffers from poor bioavailability and rapid metabolic clearance in humans, which severely restricts its systemic exposure despite encouraging in vitro outcomes [244,245]. Consequently, updated translational approaches, such as patient-derived xenografts, 3D tumor organoids, and early-phase pharmacokinetic profiling, are increasingly advocated to better simulate human biology and optimize dosing strategies [246,247,248]. Ochoa et al. [134] performed a study where they examined the combination of curcumin and resveratrol with chemotherapy in colorectal and breast cancer and exhibited improved tolerability but did not achieve statistically significant efficacy, largely due to suboptimal dosing and bioavailability challenges.

Another critical barrier lies in the standardization and reproducibility of botanical extracts. Unlike synthetic drugs, natural products are inherently complex mixtures whose chemical profiles can vary based on geographic origin, harvest season, and extraction protocols [249]. This variability undermines consistency in experimental results and complicates regulatory approval [250,251]. To address this, the ConPhyMP guidelines have been introduced to promote standardized practices in botanical authentication, chromatographic techniques (including fingerprinting), and quantification of key marker compounds [252]. Despite widespread recognition of these standards, many research laboratories still lack the capacity or standardized protocols to implement them fully, resulting in ongoing discrepancies between batches and studies.

Technological advances in nanocarrier design, including NPs and liposomes, hold great promise for overcoming pharmacokinetic limitations [253]. Novel platforms such as erythrocyte membrane-coated NPs and glycoprotein-targeted mesoporous silica systems have demonstrated improved drug stability, evasion of immune surveillance, and controlled release in tumor-specific environments (i.e., pH-sensitive or enzyme-triggered), showing efficacy in lung cancer models [24,245,254,255,256]. Liposomal formulations incorporating curcumin and paclitaxel via inhalation have likewise induced selective apoptosis in NSCLC models while sparing normal lung tissue [257]. These developments illustrate how nanotechnology can enhance both the delivery and therapeutic index of bioactive–chemotherapy combinations [258,259].

Combining natural compounds with immunotherapy is another emerging frontier [260]. Certain phytochemicals act as immune checkpoint modulators [261]; for example, apigenin downregulates PD-L1 expression and berberine promotes PD-L1 degradation, thereby enhancing cytotoxic T-cell activity [254]. EGCG has been shown to prevent chemotherapy-induced upregulation of PD-L1 via the JAK2/STAT1 signaling axis, preserving effector CD8+ T-cell function in models of resistant lung cancer [262]. Moreover, co-administration of apigenin with anti-PD-1 therapy in murine Lewis lung carcinoma models enhanced T-cell infiltration and tumor regression, suggesting that phytochemicals may effectively augment the immune effects of checkpoint inhibitors [254].

Personalized oncology further expands the potential of these combined strategies by tailoring treatments based on individual molecular and microbiome profiles [263,264]. The gut microbiota plays a pivotal role in modifying drug metabolism and immune responses; microbial enzymes can convert certain phytochemicals into active or inactive metabolites, influencing therapeutic efficacy and toxicity [265,266]. Consequently, microbiome-aware drug formulation, incorporating probiotics, prebiotics (i.e., by nutrition), or microbiome-preserving nanoparticles, could improve bioavailability and reduce variability across patient populations [267,268]. Similarly, tumor profiling that includes PD-L1 status, microsatellite instability, and tumor mutational burden could guide the selection of optimized bioactive–immunotherapy combinations, supporting more personalized and potentially effective regimens [269,270,271].

Furthermore, theranostic nanoparticle systems that co-encapsulate diagnostic imaging agents and therapeutic payloads offer real-time monitoring of drug distribution and response [272]. These smart carriers can adjust release kinetics based on imaging feedback and pharmacodynamic markers, forming the basis of closed-loop therapeutic systems that personalize dosing and minimize off-target toxicity [273,274].

Taken together, the current challenges and future perspectives in integrating natural bioactives into cancer therapy can be summarized by three key priorities: 1) enhancing translational predictability via advanced human-relevant models and robust pharmacokinetic data; 2) ensuring reproducibility and regulatory compliance through standardized botanical preparations like those recommended by ConPhyMP; and 3) advancing precision therapy using smart nanocarriers capable of delivering synergistic bioactive and chemotherapeutic agents in an immunologically informed, patient-specific manner [275]. These comprehensive strategies have the potential to transform preclinical findings into clinically impactful treatments.

## 7. Conclusions

In the context of growing therapeutic resistance, natural bioactive compounds have demonstrated substantial potential to influence both intrinsic and microenvironment-driven mechanisms that allow cancer cells to survive, adapt, and evade treatment. Their multitarget nature, encompassing effects on cell death regulation, stress responses, immune modulation, and stromal interactions, positions them as ideal candidates for complementary therapy. Unlike single-target synthetic agents, these compounds can act on various levels simultaneously, addressing the complexity of resistance in a more holistic way.

This review has highlighted key chemical families, such as polyphenols, alkaloids, terpenoids, and marine-derived metabolites, that show functional relevance in resistance models. The integration of these agents with conventional chemotherapy, supported by advances in delivery systems and pharmacokinetic improvements, may help overcome the limitations of current treatment protocols. However, to move from experimental promise to clinical reality, several challenges remain. These include the need for standardized extracts, consistent bioavailability, and robust clinical trial data.

Ultimately, harnessing the therapeutic potential of natural bioactives requires a transdisciplinary approach. Collaboration between molecular researchers, pharmacologists, clinicians, and nutriologists is essential to unlock new therapeutic pathways and provide more personalized, effective, and less toxic options for cancer patients. Natural bioactives do not replace conventional therapy, but they may be key allies in redefining resistance and improving long-term outcomes.

## Abbreviations

ABC	ATP-binding cassette
ABCB1	ATP-binding cassette sub-family B member 1
Akt	protein kinase B
AMPK	adenosine monophosphate-activated protein kinase
Arg-1	arginase 1
ATP	adenosine triphosphate
BAK	Bcl-2 antagonist/killer 1
BAX	Bcl-2-associated X protein
BCL	B-cell lymphoma
CAAs	cancer-associated adipocyte
CAFs	cancer-associated fibroblasts
CSCs	cancer stem cells
CTL	cytotoxic T lymphocyte
DNMTs	DNA methyltransferases
ECM	extracellular matrix
EGCG	epigallocatechin gallate
EGF	epidermal growth factor
EGFR	epidermal growth factor receptor
EMT	epithelial-to-mesenchymal transition
ERK	extracellular signal-regulated kinase
FAK	focal adhesion kinase
FGF	fibroblast growth factor
FX	fucoxanthin
GADD153	growth arrest and DNA damage-inducible protein 153
HDACs	histone deacetylases
HER	human epidermal growth factor receptor
HIFs	hypoxia-inducible factors
IGF-1	insulin-like growth factor-1
IL	interleukin
iNOS	inducible nitric oxide synthase
JAK	Janus kinase
KF	kahalalide F
MAPK	mitogen-activated protein kinase
MCL	myeloid cell leukemia
MDR	multidrug resistance
MDR1	multidrug resistance protein 1
MDSCs	myeloid-derived suppressor cells
MHC	major histocompatibility complex
MMPs	matrix metalloproteinases
mTOR	mammalian target of rapamycin
mTORc	mammalian target of rapamycin complex
NPs	nanoparticles
NF-κB	nuclear factor-kappa B
NSCLC	non-small-cell lung cancer
P-gp	P-glycoprotein
PD-1	programmed cell death protein 1
PD-L1	programmed cell death 1 ligand 1
PDGF	platelet-derived growth factor
PI3K	phosphoinositide 3-kinase
Tregs	regulatory T cells
ROS	reactive oxygen species
RTK	receptor tyrosine kinase
STAT	signal transducer and activator of transcription
TAMs	tumor-associated macrophages
TGF-β	transforming growth factor beta
TME	tumor microenvironment
TNF-α	tumor necrosis factor alpha
TNF-β	tumor necrosis factor beta
VEGF	vascular endothelial growth factor
Wnt	wingless-type MMTV integration site family
